# Using simulation to explore the impact of device design on the learning and performance of peripheral intravenous cannulation

**DOI:** 10.1186/s41077-019-0118-5

**Published:** 2019-11-21

**Authors:** Bronwyn Reid-McDermott, Maryanne Browne, Dara Byrne, Paul O’Connor, Emily O’Dowd, Chloe Walsh, Caoimhe Madden, Sinéad Lydon

**Affiliations:** 1 Irish Centre for Applied Patient Safety and Simulation, School of Medicine, National University of Ireland Galway, Galway, Ireland; 2 School of Psychology, Trinity College Dublin, Dublin 2, Ireland; 3 Discipline of General Practice, School of Medicine, National University of Ireland Galway, Galway, Ireland; 4 School of Medicine, National University of Ireland Galway, Galway, Ireland

**Keywords:** Medical device, Device design, Behavioral fluency, Learning, Performance, Peripheral intravenous cannulation, Peripheral intravenous catheterization, Cannulation, Generalization, Transfer

## Abstract

**Background:**

The design of medical devices impacts upon the performance of healthcare professionals and patient safety. However, multiple devices serving the same function are often available. The purpose of this study was to use simulation as a means of examining the impact of differences in device design on (1) learning of, or attainment of behavioral fluency in, peripheral intravenous cannulation (PIVC); and (2) the generalization, or transfer, of learning on one device to performance of PIVC using an untrained device.

**Methods:**

A total of 25 final cycle medical students participated in this study which used a randomized two-group design. Participants were randomly assigned to learn PIVC using either a closed PIVC device (a single device which consists of an intravenous cannula with a pre-attached extension tube; *n* = 14) or an open PIVC device (a two-piece device made up of an intravenous cannula and a separate extension tube which is attached following insertion of the cannula; *n* = 11). Task analyses were developed for the performance of PIVC using each device. Subsequently, simulation-based fluency training was delivered to both groups using their assigned PIVC device, and continued for each participant until the fluency criterion was achieved. Following achievement of fluency, participants were asked to perform PIVC using the untrained device (i.e., the PIVC device that they had not been trained on).

**Results:**

All participants in both groups met the fluency criterion, and no significant differences were observed in the number of trials or total training required by groups to achieve fluency. Participants in both groups improved significantly from baseline (*M* = 11.69) to final training trial (*M* = 100). However, a significant decrement in performance (*M* = 81.5) was observed when participants were required to perform PIVC using the untrained device.

**Conclusions:**

Participants achieved fluency in PIVC regardless of the device used. However, significant decrements in performance were observed when participants were required to perform PIVC using a novel device. This finding supports the need for careful consideration of devices purchased and supplied in the clinical setting, and the need for training prior to the introduction of novel devices or for new staff members.

## Background

It is well recognized that the design of equipment and medical devices impacts upon the performance of healthcare professionals and the safety of patients [1–3]. Errors can arise for a variety of reasons including poor engagement between those designing medical devices and those who must use these in practice, insufficient training in the use of devices, device failure, user error, inappropriate use or applications of a device, or inadequate device servicing or maintenance [1, 4–6].

The safe use of medical devices by healthcare professionals is also compromised by the diversity of devices available in the clinical environment which serve the same function [7, 8]. For instance, it is common for hospital trusts in the UK to have more than 30 different infusion pump devices [7]. Similarly, substantial variance in the design of resuscitation equipment has also been observed in UK hospitals [9]. This variety of devices creates a considerable opportunity for errors, and patient harm [8]. Healthcare professionals who work across various locations, or are new to an organization, may fail to identify or recognize crucial differences in device design [5] which can negatively impact on technique and performance.

While consistency and standardization is recognized as an important means of ensuring the safe usage of medical devices and equipment [2, 8, 10], little research has examined the impact of differences in device design on procedural skill learning or the accuracy of performance of a particular procedural skill using a novel device. The use of simulation is one means of assessing the use, or adequacy, of equipment and devices in order to inform appropriate training or education initiatives [11–13].

The purpose of this study was to examine the impact of differences in device design on (1) learning, or attainment of behavioral fluency in, a targeted procedural skill; and (2) generalization, or transfer, of learning on one device to performance of the same skill using an untrained device. Of key interest was the quantification of any difference in performance during the assessment of generalization of learning to a novel device, and the examination of the nature of any errors that were found to occur during this process.

In order to address these research questions, we enrolled final cycle medical students into a research study that involved the delivery of fluency training in peripheral intravenous cannulation (PIVC), also referred to as peripheral intravenous catheterization. Fluency training has been demonstrated previously to be effective in teaching core procedural skills at undergraduate and postgraduate medical levels [14, 15]. Peripheral intravenous cannulas are the most commonly used invasive medical device in hospital settings [16], and are used for therapeutic purposes such as administration of medications, fluids, and/or blood products as well as blood sampling. Potential complications associated with their use include phlebitis or thrombophlebitis, bloodstream infections, and infections at the site [16]. Appropriate PIVC technique is crucial for reducing the frequency of complications [17, 18], yet disparities in PIVC-related training have been reported [18]. In the Irish health service, multiple different PIVC devices are available for use across hospitals and within hospital groups across which physicians may work [19]. For the purpose of this study, we identified two of the most commonly used devices and explored the impact of device design on attainment of fluency and generalization of learning to performance using an untrained PIVC device.

## Method

### Experimental design and setting

**Fig. 1:**
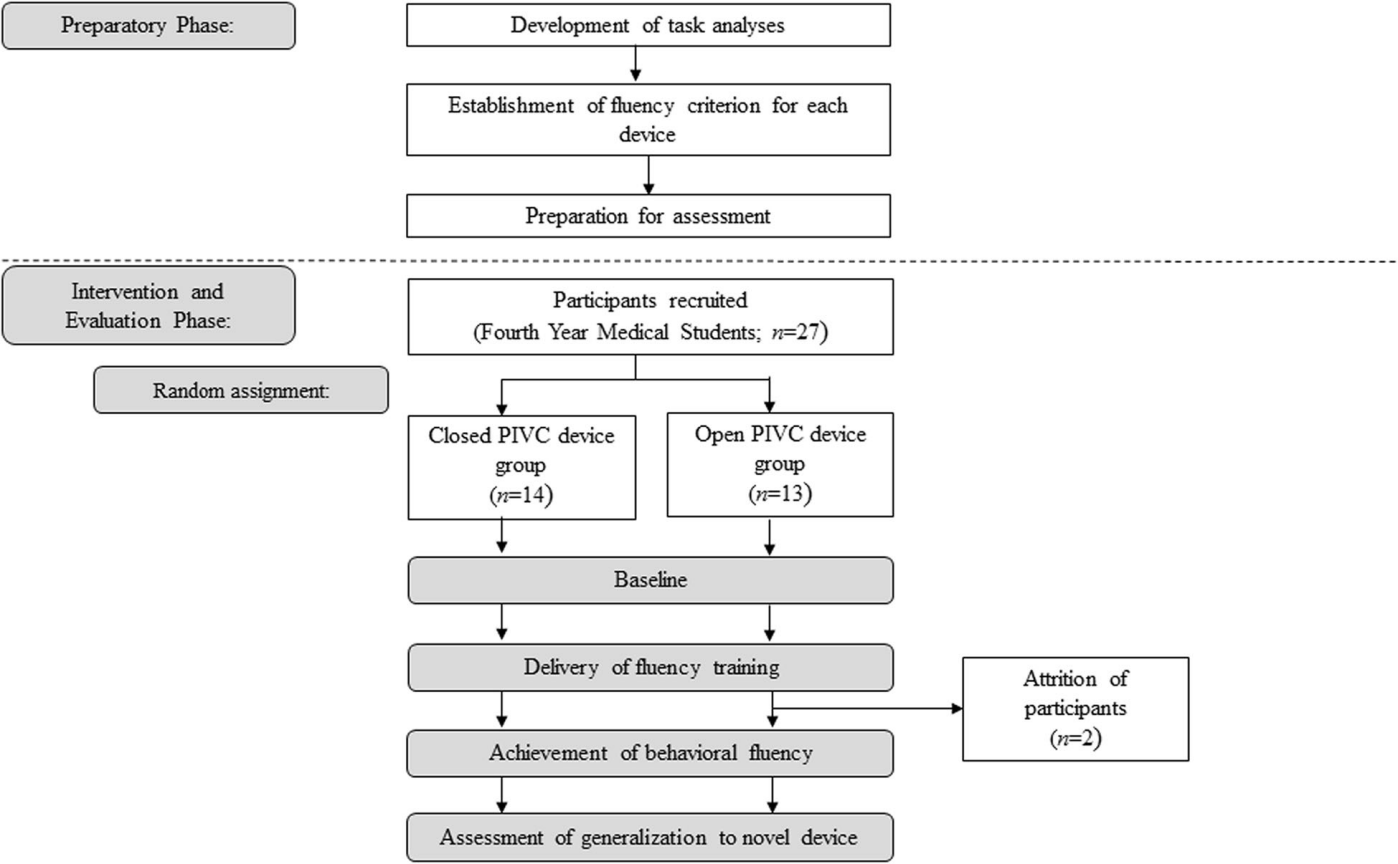
Flow diagram depicting the activities occurring during the phases of this study for intervention and control groups. *PIVC* peripheral intravenous cannulation

This study used a randomized two-group design. Fourth-year medical students were randomly assigned to complete the simulation-based fluency training using one of two different PIVC devices available for use in Irish hospitals. Within-subject analysis was used to monitor the performance of participants in both groups throughout the intervention as they worked to achieve fluency. An overview of the different phases of the study is provided in Fig. 1.

All phases of the study were carried out in the National University of Ireland Galway’s simulation laboratory.

### Ethical approval

Ethical approval was obtained from the National University of Ireland Galway’s Research Ethics Committee (Ref: 17-Sep-15).

### Participants

Convenience sampling [20] was used to recruit 27 fourth-year medical students who were subsequently randomly assigned to complete simulation-based fluency training using either the closed PIVC device or the open PIVC device. Simple randomization was employed [21], and achieved by having participants draw a small piece of paper from a hat that contained multiple pieces of paper in two different colors. Group assignment was then based on the color of the paper each participant had drawn. All participants had received previous training within the medical school on venipuncture, but had not received prior training on PIVC.

### Materials

#### Simulator

The ‘Limbs and Things’ ante-cubital fossa (ACF Pad Venipuncture Simulator, Limbs and Things, Bristol, UK) pad was used to simulate the vessels contained within the antecubital fossa of the arm and/or dorsum of the hand. This pad was attached to the non-dominant arm of the assessor to allow for a tourniquet to be applied during performance of PIVC, and was connected to a fluid bag containing mock blood to simulate blood flow (Mock Blood Giving Set, Limbs and Things, Bristol, UK).

#### Consumables

Consumables provided for the performance of PIVC using each of the two devices are presented in the corresponding task analyses (see Additional file 1: Closed PIVC device task analysis and Additional file 2: Open PIVC device task analysis). The consumables used were the same as those typically made available for performing PIVC within Irish hospitals. The primary difference between consumables for the two groups was the type of PIVC device used, either (1) a closed PIVC device or (2) an open PIVC device. Other variability was deliberately constrained by ensuring needles were of the same gauge and extensions sets had the same number of ports.

##### Closed PIVC device

A closed PIVC device is a single device which consists of an intravenous cannula with a pre-attached extension tube [22]. The closed PIVC device used in this study was BD Nexiva Closed IV Catheter System with Single Port.

##### Open PIVC device

An open PIVC device is made up of two component parts: an intravenous cannula which is inserted into the vein using a trocar technique and an extension tube which is attached to the inserted cannula following removal of the stylet [23]. The two components of the open device used in this study were the B. Braun Introcan Safety IV Catheter and BD Neutraclear 1-Way Extension Tubing.

### Procedure

#### Preparatory phase

There were three stages to the preparatory phase.

##### Development of the task analysis

The target behavior in this study was PIVC. Task analyses were developed for the performance of PIVC on both the closed and open device (see Additional file 1: Closed PIVC device task analysis and Additional file 2: Open PIVC device task analysis, respectively). These task analyses were developed in accordance with best practice and convention within applied behavior analysis (a scientific discipline concerned with changing socially significant behavior through the consideration of empirically established principles of behavior [24]) for developing task analyses [25, 26]. This process involves identifying all of the distinct behaviors that comprise completion of a ‘task’ appropriately and determining their sequence [26]. Subsequently, each distinct behavior, or task analysis step, is operationally defined to specify the physical movements involved in, and materials required for, correctly completing that step [27]. This careful and clear definition of each step is essential for ensuring accurate and reliable recording of the behavior. In order to ensure the validity of the task analyses, these were developed following a review of extant teaching materials, hospital guidelines, and manufacturer recommendations. Each step in the task analyses was operationally defined and specified both the physical movements and materials involved [14].

The initial task analyses were reviewed and refined by two subject matter experts; a clinical nurse specialist, who had over 25 years of experience in teaching PIVC and venipuncture, and a hematology nurse working as a clinical consultant with 8 years of experience. The revised task analyses were further reviewed for face validity by other healthcare educators and senior doctors (*n* = 3), by psychologists with expertise in the development of task analyses and operationalization of behavior (*n* = 2), and by lay-persons (*n* = 3) to ensure the instructions and language used were clear and accessible.

##### Establishment of the fluency criterion

The fluency criterion is a benchmark representing expert, or fluent, performance that learners work to achieve during training. The fluency criterion for performance of the target behavior using each PIVC device was determined separately, in both instances by having an expert perform the behavior with complete accuracy on three occasions and using the median duration of these performances to establish an appropriate fluency criterion. Within the subsequent fluency training, participants were required to complete the task with 100% accuracy on two successive trials, and without exceeding the expert’s median duration by more than 10%, in order to be deemed fluent. The criterion rate was set at 5 min, 14 s for the closed PIVC device and 5 min, 5 s for the open PIVC device.

##### Preparation for assessment

Data recording sheets were developed that allowed observers to document both the percentage accuracy and duration associated with participants’ performance of PIVC for each trial. The role of the observers was to formally assess participants’ performances for the purposes of research data collection and to support peer assessors (i.e., a participant in the study who observed another participant perform the target behavior, assessed their performance, and provided corrective feedback using the task analysis as a guide) in the delivery of specific corrective feedback following performances. Observers (*n* = 5) were trained in the appropriate assessment of the accuracy and duration of PIVC performance prior to beginning data collection. Videos of trials showing performances of 100% accuracy within time-frame were developed to show the break-down of steps and ensure assessors were familiar with both the task itself and the recording of data before trials began. Observers were allowed multiple attempts to practice trials using both PIVC devices, document the performance of others using recording sheets, and to give and receive corrective feedback. The marking of recording sheets during training was reviewed by the lead researcher (BRM) in order to ensure each observer’s data were accurate and corrective feedback was provided where necessary.

#### Intervention and evaluation phase

There were three stages to the intervention phase.

##### Assessment of baseline performance

Following random assignment, participants were asked to carry out PIVC ‘to the best of their ability’ using their assigned device (i.e., either open or closed). Participants were first habituated to the simulator and were given the opportunity to inspect the device and other materials provided, as per best practice in healthcare simulation research [28]. Observers gave no further instruction on how to use any of the consumables provided. Participants were informed that the accuracy and speed of their performance would be assessed by an observer. For each trial in the baseline and later phases of the intervention, the observer indicated when they were starting the timer, at a time when the participant was comfortably situated at the desk but had engaged in no physical movements related to the performance of PIVC. The timer was stopped when the participant had performed the final step of the related task analysis or indicated verbally that they were finished. Upon completion of the baseline trial, participants were given the task analysis for their assigned device but were not provided with corrective feedback.

##### Fluency training

In this stage, participants attended a series of training sessions at the simulation laboratory. During the first training session, participants were assigned to groups of two to three and it was explained that during each trial (defined as one performance of the behavior using the simulator), one participant would act as the ‘learner’ and the other participants would act as the ‘peer assessors,’ using the recording sheet, and that participants would subsequently take turns in each role. It was explained that the goal was to practice until they had achieved behavioral fluency (defined as 100% accuracy across two consecutive trials within the criterion rate for their assigned device—5 min, 14 s for the closed PIVC device and 5 min, 5 s for the open PIVC device).

Following each learning trial, the peer assessors provided corrective feedback using the task analyses and recording sheet on the accuracy of the trial. A trained observer was also stationed with each group and provided input and support for any additional or uncertain elements following the initial peer feedback. The opportunity to practice incorrect steps was provided at the end of each trial. One physician was present at every training session to answer any technical and/or clinical questions that arose.

Throughout the intervention phase, standard celeration charts (SCCs), produced using Chartlytics© software, were used to graph and monitor the progress of each participant and identify any potential need for additional or modified training methods. SCCs are a core element of precision teaching, an educational framework often used alongside fluency training [27, 29]. SCCs depict the frequency of a target behavior and are widely used by researchers or educators to facilitate determination of whether learning is progressing under current instructional conditions or whether a change in, or additional, instructional techniques is required [30]. Participants were shown their individual, up-to-date graph at the beginning of each training session. For each participant, training continued until they had achieved the fluency criterion.

##### Assessment of generalization of learning to untrained device

In order to assess the generalization, or transfer, of the target behavior to an untrained PIVC device, participants from both groups were asked to perform PIVC to the best of their ability using the PIVC device that was not used during their fluency training (i.e., participants who had learned to perform PIVC using the open device were now assessed on their performance using the closed PIVC device and participants who had learned to perform PIVC using the closed device were now assessed on their performance using the open PIVC device), subsequent to achieving fluency. This assessment was conducted in the same manner as baseline testing and the relevant task analysis was not made available to these individuals until the study was completed. The accuracy of participants’ performance, evaluated as per the relevant task analysis, was the focus of this assessment.

### Data analysis

SPSS version 24 (Armonk, NY: IBM Corp) was used for data entry and analysis. To examine for any differences in the ease or rapidity of learning PIVC using the closed and open PIVC devices, two independent *t* tests were used to analyze whether the number of trials to completion or total training time differed by group. To control for this multiple testing, a Bonferroni correction was applied, and alpha was set at .025. In order to further assess for differences in performance among the closed PIVC device and open PIVC device groups, a mixed ANOVA was used to compare the performance of both groups across baseline, final training trial, and the generalization assessment.

## Results

### Participants

The participants were randomized into two groups. The resulting closed PIVC device group consisted of 14 fourth-year medical students (eight women, six men). Mean age was 22.77 years (*SD* = 1.24; range 21–25). The open PIVC device group consisted of 13 students. However, two participants in this group withdrew from the study and their data is not reported. Therefore, the final open group consisted of 11 fourth-year medical students (five women, six men). Mean age was 22.36 years (*SD =* 0.81; range 21–24).

### Impact of differences in device design on learning

The closed PIVC device group achieved behavioral fluency in a mean of 9.50 trials (*SD* = 2.74) and a mean total training time of 57.47 min (*SD* = 15.26). The open PIVC device group achieved behavioral fluency in a mean of 11 trials (*SD* = 2.739) and mean total training time was 69.75 min (*SD* = 18.03). Independent *t* tests showed that there was no significant difference between the groups on the number of trials taken to reach fluency, *t* (23) = − 1.36, *p* = 0.19, or in the total duration of training, *t* (23) = − 1.84, *p* = 0.08.

### Impact of differences in device design on generalization of learning

**Fig. 2:**
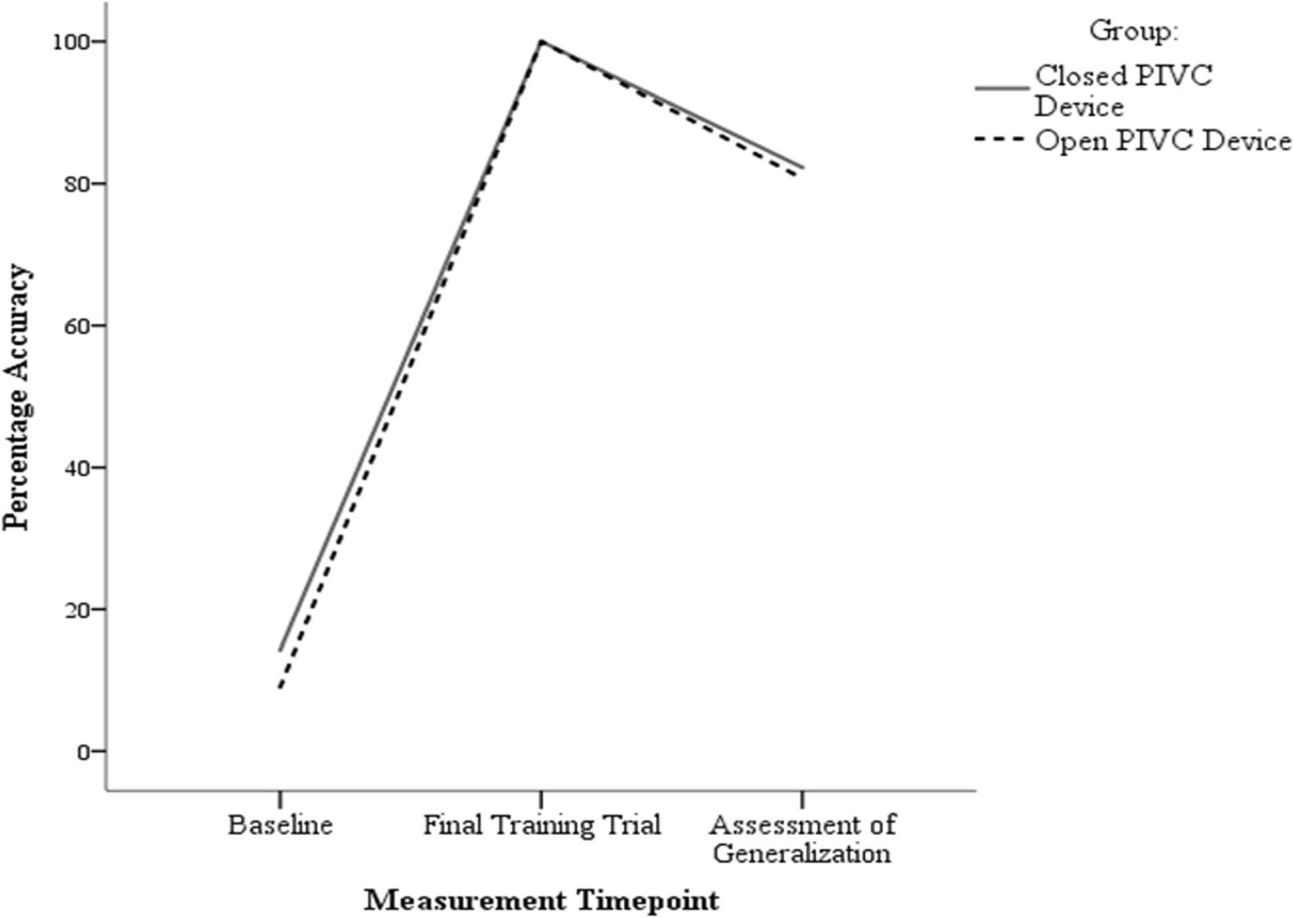
Line graph depicting the performance of both groups across baseline, final training trial, and the assessment of generalization

The performance of both groups across baseline, final training trial, and generalization to untrained device assessment is depicted in Fig. 2. A mixed ANOVA was used to assess for statistically significant differences in performance between groups and across measurement timepoints. This test revealed no discernible main effect of group, i.e., no significant differences between the performance of the two different device groups, *F*(1,23) = 1.32, *p* = .26. Similarly, the test indicated no significant interaction effect, i.e., no significant difference between the performance of the two groups at any of the different measurement timepoints, *F*(1,23) = .50, *p* = .49. However, a main effect of measurement timepoint was identified, *F*(1,23) = 734.10, *p* < .001, partial η^2^ = .97 (large effect). Bonferroni-corrected pairwise comparisons were subsequently performed in order to determine where the differences in performance lay. These pair-wise comparisons revealed that accuracy at baseline (*M* = 11.69) was significantly lower than accuracy at the final training trial (*M* = 100) and the generalization to novel device trial (*M* = 81.51), all *p*’s < 0.001. However, although performance during the generalization phase was significantly higher than performance at baseline (*p* < 0.001), it was significantly lower than performance at final training trial using the device participants had received training on (*p* < .001).

No participant in either group performed with complete accuracy on the untrained device. An examination of the errors made by participants during the generalization phase was undertaken. Common errors that occurred during the use of the closed PIVC device were a failure to loosen the safety mechanism (100% of participants), to flush and attach the bung (63.6% of participants), and to operate the pinch clamp (72.7% of participants; steps 4, 12, 13, and 21 within the task analysis; see Additional file 1: Closed PIVC device task analysis). Average accuracy when using the closed PIVC device was 80.3% (range 69–88%, M errors 5.1).

Most commonly, participants using the open PIVC device for the first time made errors relating to flushing the extension tubing during equipment set-up (92.9% of participants), placing gauze underneath the needle insertion point (50% of participants), and flushing the extension tubing once the cannula was secured (50% of participants; steps 12, 17, and 23 within the task analysis; see Additional file 2: Open PIVC device task analysis). Average accuracy when using the open PIVC device was 82.3% (range 56–96%, M errors 4.4).

## Discussion

This study used simulation as a means of examining the impact of differences in device design on learning and generalization of learning. No effect of device design was found for acquisition of fluency in a target behavior. However, significant decrements in performance were observed when participants were required to generalize their learning to a PIVC device on which they had not been trained. Errors made when using the untrained PIVC device may increase the likelihood of PIVC-related complications and infections. This finding highlights the need for careful consideration of the devices made available in clinical settings and surrounding the introduction of new devices or staff members in clinical settings.

There was no apparent impact of device design on acquisition of fluency in completing PIVC. Participants in both groups required a mean of 10.2 trials to achieve fluency in the target behavior. These data underscore the necessity of dedicated procedural skills training and the insufficiency of traditional approaches to skills training such as ‘See One, Do One, Teach One’ [31]. Fluency training may offer one means of ensuring effective skill development during medical school. Previous research [14, 15] has also demonstrated the efficacy of fluency training for producing a high level of performance, and learning that is stable (i.e., persists in the presence of distraction) [32], retains (i.e., persists over time following the cessation of training) [32], and generalizes to the clinical setting. The ability of healthcare professionals to perform core procedural skills fluently is likely to positively impact on patient safety [33]. Future research which considers how best to establish fluency or proficiency in core procedural skills within busy medical school curricula would be of much interest.

The performance decrements observed when participants were required to transfer their learning to a novel, untrained PIVC device are of note. Although participants were performing with complete accuracy at the conclusion of training, percentage accuracy deteriorated to a mean of 81.5 (SD = 8.3) when participants were asked to use the PIVC device on which they had not been trained and critical errors were made. Correct performance of PIVC is essential for reducing the frequency of complications [17, 18]. Issues such as not flushing the extension tubing prior to use, not applying gauze to reduce the risk of blood spillage, failing to loosen the safety mechanism on the closed PIVC device, and not closing the pinch clamp on the closed PIVC device could contribute to an increased risk of blood exposure for the healthcare practitioner and of PIVC-related complications for the patient such as phlebitis or thrombophlebitis, bloodstream infections, air embolism and infections at the cannulation site [16].

In addition to the negative impact of poor performance upon patients, uncertainty regarding the appropriate use of a device has been found to cause stress for healthcare providers [10]. These data support the need for simulation-based training upon the introduction of novel devices in the clinical setting or at induction for new staff members [23, 24]. Future research that further explores the impact of device design on performance among varying professional groups would be of use in strengthening the argument or rationale for dedicating resources to ensure all staff, even temporary or agency staff, are proficient in the use of medical devices necessary for the safe and effective performance of core procedural skills.

### Limitations

There are a number of limitations to this study that should be acknowledged. First, the lack of formal consideration of sample size in advance of the conduct of the study and reliance on non-random, convenience sampling may be criticized. Second, the inclusion of medical students as participants, rather than experienced healthcare providers, when considering the impact of device design on learning and performance may also be questioned. However, using medical students was considered appropriate as then participants had minimal knowledge of PIVC and little, or no, exposure to the different PIVC devices under examination. Further, previous research on fluency training suggests that post-fluency training, learners perform with higher levels of accuracy as compared to untrained, but more senior, peers who have greater degrees of clinical experience [14, 15]. Third, data relating to the reliability of assessment (i.e., interobserver agreement data) were not collected during the study. It may be noted that observers were performing reliably during training as part the ‘preparation for assessment’ phase. However, data were not collected beyond this phase. Finally, data were not collected regarding the participants’ perceptions of the PIVC devices’ ease of use. Such data have been gathered in previous research and have revealed perceived differences in the ease of use of open and closed PIVC devices [23]. In our study, engagement with participants’ regarding their experiences using the two devices may have elucidated the errors occurring during the generalization phase and informed future training endeavors.

### Considerations for future research

A number of considerations for future research may be offered. First, the impact of acquiring fluency in one target behavior on the acquisition of fluency in another target behavior, or the development of fluent performance on an untrained device, should be considered. In the current study, participants required a mean of 10.2 (SD = 2.8) trials to achieve fluency across the two PIVC devices. Future research could explore whether the trials, and training time, taken to produce fluency in the performance of a procedural skill using an untrained device, or in other related trocar skills such as lumbar puncture and intraosseous cannulation, are reduced. Given that the accuracy of performance during the generalization assessment remained significantly higher than performance at baseline, it might be expected that fewer training trials would be required to produce fluency in these instances. However, research which empirically investigates this possibility would be of much interest.

Second, future research could usefully examine whether only the provision of detailed task analyses to the participants, describing the appropriate performance of a procedural skill using a specified device, may be sufficient to produce safe and effective performance of skills using novel devices in the absence of training. Task analyses make the knowledge of an expert, or experts, explicit for a learner [34]. In addition to task analyses’ use for facilitating an instructor’s assessment of performance or learning [35], task analyses make clear for a learner, or novice, what behavior is required or expected in the performance of a particular skill [14]. In the current study, participants completed a mean of 4.72 steps incorrectly during the assessment of generalization. It is possible that the provision of a relevant task analysis, in the absence of further training, might be sufficient to clarify appropriate performance of these steps for participants and improve performance on the novel device. This would constitute a minimally expensive means of ensuring appropriate performance and patient safety. However, research data is first required to elucidate the impact of a task analysis provided in the absence of further training on performance.

Finally, data collected in the current study suggest the need for greater consideration of the devices purchased and supplied in clinical settings and the introduction of novel devices. Previous research has identified that it is common for multiple devices serving the same function to be made available to healthcare providers [7, 9] and that this can negatively impact upon performance [7, 8]. The current study has also demonstrated that performance decrements occur when participants are required to use novel devices on which they have not been trained. There are a substantial number of factors that must be considered during the selection and purchasing of devices including their safety for the patient and likelihood of contributing to positive outcomes, the safety of their use for the healthcare provider, and the cost [23]. However, ease of use by healthcare providers should also be considered [23]. The decrements in performance observed in the current study suggest that the impact of device on performance and a potentially increased occurrence of errors should be acknowledged and contribute to purchasing decisions.

## Conclusions

There was no discernible impact of device design on learning of, or the attainment of behavioral fluency in, PIVC. However, device design appeared to have an impact on subsequent performance, and significant decrements in the accuracy of PIVC performance were observed when participants were required to perform PIVC using a commonly available but untrained PIVC device. These data emphasize the need for the careful consideration of the devices that are made available in clinical settings, and for appropriate simulation-based training to be provided upon the introduction of a novel device, or new staff members, into the clinical setting.

## Supplementary information

**Additional file 1.** Closed PIVC device task analysis.

**Additional file 2.** Open PIVC device task analysis.

## Data Availability

The datasets used and/or analyzed during the current study are available from the corresponding author on reasonable request.

## References

[1] Lawton R, McEachan RRC, Giles SJ, Sirriyeh R, Watt IS, Wright J (2012). Development of an evidence-based framework of factors contributing to patient safety incidents in hospital settings: a systematic review. BMJ Qual Saf.

[2] Zhang J, Johnson TR, Patel VL, Paige DL, Kubose T (2003). Using usability heuristics to evaluate patient safety of medical devices. J Biomed Inform.

[3] Leape LL, Brennan TA, Laird N, Lawthers AG, Localio AR, Barnes BA, Hebert L, Newhouse JP, Weiler PC, Hiatt H (1991). The nature of adverse events in hospitalized patients: results of the Harvard medical practice study II. N Engl J Med.

[4] Money AG, Barnett J, Kuljis J, Craven MP, Martin JL, Young T (2011). The role of the user within the medical device design and development process: medical device manufacturers’ perspectives. BMC Med Inform Decis Mak.

[5] Balka E, Doyle-Waters M, Lecznarowicz D, Fitzgerald JM (2007). Technology, governance and patient safety: systems issues in technology and patient safety. Int J Med Inform.

[6] Blandford A, Furniss D, Vincent C (2014). Patient safety and interactive medical devices: realigning work as imagined and work as done. Clin Risk.

[7] Lowe C (2006). Accidents waiting to happen: the contribution of latent conditions to patient safety. BMJ Qual Saf.

[8] Rodkin S (2007). Purchasing for safety: standardization in intravenous equipment. Br J Nurs.

[9] Hussey S, Ryan C, Murphy B (2004). Comparison of three manual ventilation devices using an intubated mannequin. Arch Dis Child Fetal Neonatal.

[10] Reiling J (2006). Safe design of healthcare facilities. BMJ Qual Saf.

[11] Lighthall GK, Poon T, Harrison TK (2010). Using in situ simulation to improve in-hospital cardiopulmonary resuscitation. Jt Comm J Qual Saf.

[12] Sherwin J (2012). More than make believe: the power and promise of simulation. Biomed Instrum Technol.

[13] Gaba DM (2004). The future vision of simulation in health care. BMJ Qual Saf.

[14] Lydon S, Burns N, Healy O, O'Connor P, Reid-McDermott B, Byrne D (2017). Preliminary evaluation of the efficacy of an intervention incorporating precision teaching to train procedural skills among final cycle medical students. BMJ Simul Technol Enhanced Learn.

[15] Lydon S, Reid McDermott B, Ryan E, O'Connor P, Dempsey S, Walsh C, Byrne D (2019). Can simulation-based education and precision teaching improve paediatric trainees’ behavioural fluency in performing lumbar puncture?. A pilot study BMC Med Educ.

[16] Zingg W, Pittet D (2009). Peripheral venous catheters: an under-evaluated problem. Int J Antimicrob Agents.

[17] Aziz AM (2009). Improving peripheral IV cannula care: implementing high-impact interventions. Br J Nurs.

[18] Alexandrou E, Ray-Barruel G, Carr PJ, Frost S, Inwood S, Higgins N, Lin F, Alberto L, Mermel L, Rickard CM (2015). International prevalence of the use of peripheral intravenous catheters. J Hosp Med.

[19] Health Service Executive (2010). National Clinical Policy and Procedural Guideline for Nurses and Midwives undertaking Peripheral Cannulation in Adults.

[20] Gelo O, Braakmann D, Benetka D (2009). Quantitative and qualitative research: beyond the debate. Integr Psychol Behav Sci.

[21] Beller EM, Gebski V, Keech AC (2002). Randomisation in clinical trials. Med J Aust.

[22] McNeill EE, Hines NL, Phariss R (2009). A clinical trial of a new all-in-one peripheral-short catheter. JAVA.

[23] González López JL, del Palacio EF, Martí CB, Corral JO, Portal PH, Vilela AA (2009). COSMOS—a study comparing peripheral intravenous systems. Br J Nurs.

[24] Baer DM, Wolf MM, Risley TR (1987). Some still-current dimensions of applied behavior analysis. J Appl Behav Anal.

[25] Cooper JO, Heron TE, Heward WL (2017). Applied behavior analysis.

[26] Steege MW, Mace FC, Perry L, Longenecker H (2007). Applied behavior analysis: beyond discrete trial teaching. Psychol Sch.

[27] Kubina RM, Yurich KK (2012). Precision teaching book.

[28] Cheng A, Kessler D, Mackinnon R, Chang TP, Nadkarni VM, Hunt EA, Duval-Arnould J, Lin Y, Cook DA, Pusic M, Hui J, Moher D, Egger M (2016). Auerbach, INSPIRE Reporting guidelines for health care simulation research: extensions to the CONSORT and STROBE statements. Adv Simul.

[29] Ramey D, Lydon S, Healy O, McCoy A, Holloway J, Mulhern T (2016). A systematic review of the effectiveness of precision teaching for individuals with developmental disabilities. Rev J Autism Dev Disord.

[30] Calkin AB (2005). Precision teaching: the standard celeration charts. Behav Anal Today.

[31] Rodriguez-Paz J, Kennedy M, Salas E, Wu AW, Sexton JB, Hunt EA, Pronovost PJ (2009). Beyond “see one, do one, teach one”: toward a different training paradigm. BMJ Qual Saf.

[32] Binder C (1996). Behavioral fluency: evolution of a new paradigm. Behav Anal.

[33] Scalese RJ, Obeso VT, Issenberg SB (2008). Simulation technology for skills training and competency assessment in medical education. J Gen Intern Med.

[34] Allery L (2009). How to… teach practical skills. Educ Prim Care.

[35] Gallagher AG (2012). Metric-based simulation training to proficiency in medical education: what it is and how to do it. Ulster Med J.

