# Supplementary material for: Using simulation to explore the impact of device design on the learning and performance of peripheral intravenous cannulation
Source: Adv Simul (Lond). 2019 Nov 21;4:27. doi: 10.1186/s41077-019-0118-5 (PMC6868858; doi:10.1186/s41077-019-0118-5)

CLOSED PIVC DEVICE TASK ANALYSIS

**Consumables Required:**	- Alcohol hand rub<br>- Blood collection tray with sharps bin<br>- 70% alcohol wipes<br>- Non-sterile gloves<br>- Disposable tourniquet<br>- Cleansing swab (70% alcohol and 2% chlorhexidine)<br>- Pre-prepared flush (10ml syringe)<br>- Single port 20G BD Nexiva closed cannulation system<br>- Needlefree connector device (bung)<br>- IV dressing<br>- Clinical waste bin

	Task	Operational Definition
1	**Perform hand hygiene with gel**	1. Participant applies alcohol gel to the palm of one hand and rubs both palms together so that gel is evenly distributed.<br>2. Rubs the back of each hand with the palm of the other hand with fingers interlaced.<br>3. Rubs palm to palm with fingers interlaced.<br>4. Rubs with back of fingers to opposing palms with fingers interlocked for each hand.<br>5. Encloses the thumb of one hand within a fist of the other and rotates for both hands consecutively.<br>6. Rubs tips of fingers in opposite palm in a circular motion for each hand.<br>7. Rubs each wrist with opposite hand using a circular motion.<br>(see Appendix 1).
2	**Clean tray**	Participant cleans the entire surface of the tray with a 70% alcohol wipe and disposes of the wipe in general waste.
3	**Open equipment**	Participant places tourniquet and unopened cleansing swab in the tray. Participant opens the packets of the following pieces of equipment and leaves the equipment resting inside the sterile pack when placed in the tray: pre-prepared saline flush, BD Nexiva closed cannulation system, bung and IV dressing.
4	**Loosen safety mechanism of BD Nexiva closed cannulation system**	Leaving the BD Nexiva cannulation device resting in its packaging, participant holds the grey safety device in one hand and uses the other hand to hold the white finger tabs, partially sliding the stylet in and out, without removing it fully, to loosen the connection and ensure ease of removal after insertion.
5	**Perform hand hygiene with gel**	1. Participant applies alcohol gel to the palm of one hand and rubs both palms together so that gel is evenly distributed.<br>2. Rubs the back of each hand with the palm of the other hand with fingers interlaced.<br>3. Rubs palm to palm with fingers interlaced.<br>4. Rubs with back of fingers to opposing palms with fingers interlocked for each hand.<br>5. Encloses the thumb of one hand within a fist of the other and rotates for both hands consecutively.<br>6. Rubs tips of fingers in opposite palm in a circular motion for each hand.<br>7. Rubs each wrist with opposite hand using a circular motion.<br>(see Appendix 1).
6	**Apply gloves**	Participant puts a non-sterile glove on each hand.

7	**Apply tourniquet**	Participant applies the tourniquet around the patient's arm about 4 inches/10 centimeters above the proposed site. The tourniquet is applied by facing the white side towards the patient's arm, pulling on both ends of the tourniquet to fully extend the elastic strap and securing the tourniquet tightly around the patient's arm using the velcroed area.
8	**Palpate suitable vein**	Participant gently examines the veins within the antecubital fossa/dorsum of hand by touch to select an appropriate vein (springy/bouncy) for cannulation.<br>Note regarding vein selection:<br>1. Feel rather than look, ideally choose a long, straight vein that feels bouncy rather than the one that is most visible.<br>2. Start with distal veins and work proximally.<br>3. Where possible, insert the cannula in the non-dominant side for patient's convenience.
9	**Cleanse skin**	Participant opens a cleansing swab and uses it to clean the entirety of the cannulation site. Participant scrubs repeatedly downwards and then repeatedly across to create a + pattern. Participant disposes of the swab in general waste.
10	**Relax tourniquet and allow to dry**	Participants loosens the tourniquet by releasing the Velcro hold but leaving the tourniquet resting in position around the patient's arm and waits 30 seconds to allow the alcohol to dry.
11	**Unlock saline flush**	While waiting for the alcohol to dry, participant faces the tip of the pre-prepared saline syringe upwards, and applies pressure to the plunger with the cap still in place until a "click" is felt. Participant then untwists the cap of the syringe approximately ¼ of a turn and expels excess air by pressing the plunger until liquid has reached the tip of the syringe and no visible air is present.
12	**Attach bung to saline syringe**	Participant connects the bung to the tip of the pre-prepared saline syringe by holding the clear centre and connecting the orange end of the bung with a push and twist motion.
13	**Flush bung**	Participant flushes the bung with 1ml of saline and returns the pre-filled saline syringe and the attached bung to the syringe packaging.
14	**Re-apply tourniquet**	Participant re-applies tourniquet by facing the white side towards the patient's arm, pulling on both ends of the tourniquet to fully extend the elastic strap and securing the tourniquet tightly around the patient's arm using the velcroed area.
15	**Insert cannula bevel up**	Participant removes the needle cover and inserts the cannula needle into the selected vein at approximately a 30 degree angle (20 – 40 degrees range is acceptable) with the bevel (the slanted, extended opening at the tip of the needle) facing upwards.
16	**Release tourniquet upon flashback**	Upon the appearance of flashback (blood in the cannula), participant undoes the tourniquet and removes it from the patient's forearm.<br><br>If flashback is not observed upon insertion of the cannula needle, participant trials the gentle advancement and retraction of the needle. If flashback is still not observed, participant should remove the cannula completely and try again with a new cannula at an alternative site. The cannula should never be fully removed and re-inserted into the skin.
17	**Advance cannula**	Participant lowers the angle of the device slightly towards the skin and advances the cannula by very gently pushing forward the grey push tab of the device while holding the white finger grips at the end of the stylet in place until the hub is touching the skin. This can be done using both hands or using a one-handed movement.
18	**Remove stylet and apply safety**	Participant holds the pink wings of the cannula flat to the skin with one hand while using the other hand to gently pull back on the white finger grips of the stylet in one smooth straight movement, removing it from the cannula completely and allowing the needle safety to apply.<br><br>Flow of blood down the extension tubing must be observed to confirm cannula is in its place.<br><br>If blood flow is not observed, the cannula is not within the vein and should be removed.
19	**Dispose of sharp correctly**	The participant puts the removed stylet into the sharps container.

**20**	**Apply dressing**	Participant then applies the dressing:<br>1. The smallest square piece of the dressing is first placed underneath the cannula's insertion site to suspend it off the patient's skin.<br>2. The "omega" shaped piece is placed on top of the cannula ensuring the site of insertion is visible through the clear window.<br>3. One of the long strips of the dressing is used to secure the extension tubing in place by stretching the strip across the point where the extension tubing connects to the cannula.<br>4. The final strip is used to secure the remaining loose tubing to the patient's skin to prevent it from swinging or becoming caught.<br>(see Appendix 2).
**21**	**Close pinch clamp**	Participant closes the pinch clamp located along the length of the extension tubing.
**22**	**Remove vent plug**	Participant removes the clear coloured air-vent plug from the distal port of the extension tubing (opposite end of tubing to the insertion point) with a gentle pull and disposes of this in sharps.
**23**	**Attach bung to removed vent plug site**	Participant removes the white cap from the bung, leaving it attached to the pre-prepared saline syringe and taking care not to touch the sterile tip underneath. Participant then connects the bung to the extension tubing with the saline syringe attached using a push and twist motion.
**24**	**Flush tubing**	Participant opens the pinch clamp of the extension tubing and flushes the attached bung and extension tubing with 5mls of saline using push-pause-positive-pressure. This involves the participant repeatedly applying and removing pressure to the plunger of the syringe using their thumb, creating a rapid stop-start pulsating motion.<br>Participant closes the clamp of the extension tubing while maintaining hold with their thumb on the plunger syringe.<br><br>Any resistance to saline flush detected indicates the cannula is not in the vein and it should be removed.
**25**	**Clean up**	Participant removes gloves and places these and any blood stained items into clinical waste. Participant places syringe in sharps bucket. Participant places any uncontaminated waste into the general waste bin.
**26**	**Perform hand hygiene with gel**	1. Participant applies alcohol gel to the palm of one hand and rubs both palms together so that gel is evenly distributed.<br>2. Rubs the back of each hand with the palm of the other hand with fingers interlaced.<br>3. Rubs palm to palm with fingers interlaced.<br>4. Rubs with back of fingers to opposing palms with fingers interlocked for each hand.<br>5. Encloses the thumb of one hand within a fist of the other and rotates for both hands consecutively.<br>6. Rubs tips of fingers in opposite palm in a circular motion for each hand.<br>7. Rubs each wrist with opposite hand using a circular motion.<br>(see Appendix 1).

Appendices:

Appendix 1: How to handrub

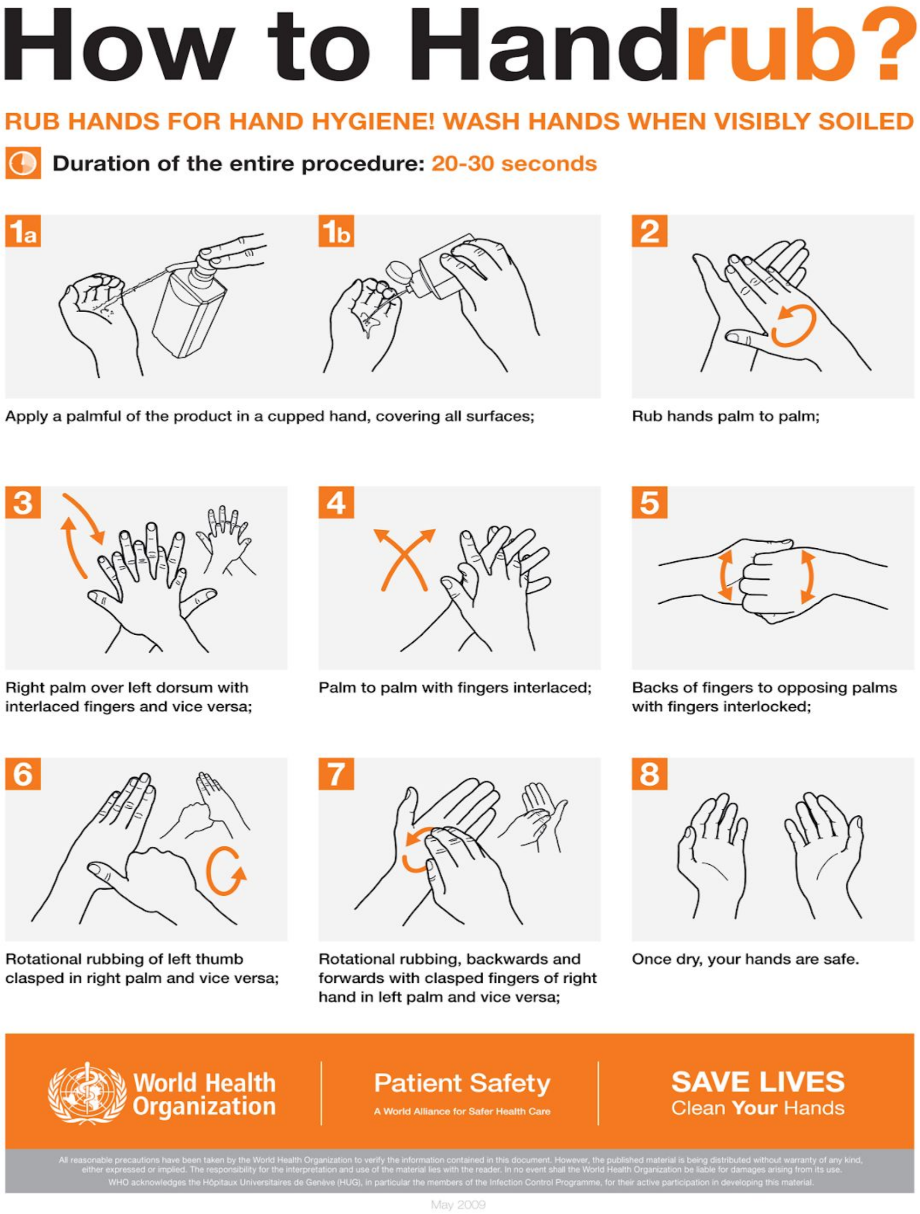

Appendix 2: How to apply a veniguard dressing

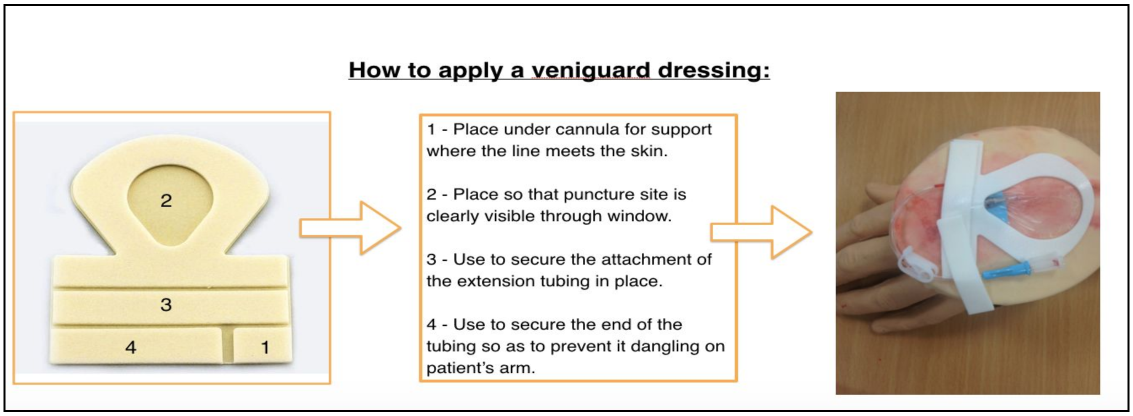

Supplement: Supplementary file 1 — Additional file 1. Closed PIVC device task analysis. [file 41077_2019_118_MOESM1_ESM.pdf]
